# A new insight on structural and some functional aspects of peri-endodermal thickenings, a specific layer in *Noccaea caerulescens* roots

**DOI:** 10.1093/aob/mcaa069

**Published:** 2020-04-16

**Authors:** Ján Kováč, Alexander Lux, Milan Soukup, Marieluise Weidinger, Daniela Gruber, Irene Lichtscheidl, Marek Vaculík

**Affiliations:** 1 Department of Plant Physiology, Faculty of Natural Sciences, Comenius University in Bratislava, Bratislava, Slovakia; 2 Institute of Botany, Plant Science and Biodiversity Centre, Slovak Academy of Sciences, Bratislava, Slovakia and; 3 Core Facility Cell Imaging and Ultrastructure Research, University of Vienna, Vienna, Austria

**Keywords:** Alpine pennycress (*Noccaea caerulescens*), apoplasmic barrier, *Arabidopsis*, cell wall, cell wall thickenings, endodermis, heavy metals, lignin, pectin, peri-endodermis, phi thickenings

## Abstract

**Background and Aims:**

Cell walls of the peri-endodermis, a layer adjacent to the endodermis in alpine pennycress (*Noccaea caerulescens*) roots, form C-shaped peri-endodermal thickenings (PETs). Despite its specific position close to the endodermis, the assumed similarity of PETs to phi thickenings in many other species, and the fact that *N. caerulescens* is a well-studied heavy-metal-hyperaccumulating plant, the PET as a root trait is still not understood.

**Methods:**

Here, we characterized PET cell walls by histochemical techniques, Raman spectroscopy, immunolabelling and electron microscopy. Moreover, a role of PETs in solute transport was tested and compared with *Arabidopsis thaliana* plants, which do not form PETs in roots.

**Key Results:**

Cell walls with PETs have a structured relief mainly composed of cellulose and lignin. Suberin, typical of endodermal cells, is missing but pectins are present on the inner surface of the PET. Penetrating dyes are not able to cross PETs either by the apoplasmic or the symplasmic pathway, and a significantly higher content of metals is found in root tissues outside of PETs than in innermost tissues.

**Conclusions:**

Based on their development and chemical composition, PETs are different from the endodermis and closely resemble phi thickenings. Contrarily, the different structure and dye impermeability of PETs, not known in the case of phi thickenings, point to an additional barrier function which makes the peri-endodermis with PETs a unique and rare layer.

## INTRODUCTION

The root cortex, the tissues between the root epidermis and pericycle, is characterized by two barrier layers: the endodermis and, if present, the exodermis. They are distinguished by cell wall modifications well known as apoplasmic barriers: Casparian bands and suberin lamellae. Cell layers between these two layers, called mesodermis or mid-cortex, form in some species a different type of wall modification called phi thickenings, named after the shape they form in cross-sections, resembling the Greek letter phi (Φ) (Russow, 1875; Guttenberg, 1968; Lux *et al.*, 2004). Generally, Casparian bands are located in primary cell walls of endo- or exodermal cells, while phi thickenings may occupy the secondary walls of mesodermal cells. Based on their location, three classes of phi thickenings can be recognized: thickenings formed in cells adjacent to the endodermis (type I), those adjacent to the root epidermis (type II), and thickenings localized between the mentioned layers but not adjacent to them (type III) (van Tieghem, 1888). Across the plant kingdom, phi thickenings are distributed in roots of various species, including both gymnosperms and angiosperms (Fernández-García *et al.*, 2014). Phi thickenings, regardless of type, are localized on radial and transverse cell walls (Mackenzie, 1979; Peterson *et al.*, 1981; Pratikakis *et al.*, 1998), but can be found also on tangential walls or around whole cells (Haas *et al.*, 1976; Gerrath *et al.*, 2002; Idris and Collings, 2015). Relatively little is known about phi thickenings formed on radial and inner tangential cell walls; as described in *Brassica oleracea*, prominent phi thickenings are localized on radial cell walls accompanied by smaller cell wall ingrowths on inner tangential walls (Lopez‐Perez *et al.*, 2007; Fernandez-Garcia *et al.*, 2009).

In the heavy metal hyperaccumulator *Noccaea caerulescens* (alpine pennycress, formerly *Thlaspi caerulescens*) the thickenings described by Zelko *et al.* (2008) are localized on radial and inner tangential cell walls of cells adjacent to the endodermis. Compared with *B. oleracea*, in *N. caerulescens* the thickenings completely occupy the inner tangential and radial cell walls and in cross-section resemble a half-moon or the letter C (van de Mortel *et al.*, 2006; Broadley *et al.*, 2007; Richau *et al.*, 2009; Aleamotu’a *et al.*, 2018). Zelko *et al.* (2008) named the cell layer in this species with formed thickenings as the ‘peri-endodermis’ or ‘peri-endodermal layer’, based on its position adjacent to the endodermis. On the contrary, some authors (Fernandez-Garcia *et al.*, 2009; Aleamotu’a *et al.*, 2018) suggest that all cortical layers with thickenings, including those in *N. caerulescens*, should be included under the term ‘phi thickenings’, even if they do not obviously have the phi shape *sensu*Russow (1875). The confusion between phi thickenings and thickenings in *N. caerulescens* is caused not only by the unclear terminology, but also by the lack of information about their structure, ontogenesis and function. According to their shape, the thickenings in *N. caerulescens* could represent a completely different group compared with common phi thickenings, as they may differ in composition, origin and development. To clearly distinguish individual thickenings, we will use the term adopted by Zelko *et al.* (2008): ‘peri-endodermal layer’ or ‘peri-endodermis’, ‘peri-endodermal cells’ or ‘peri-endodermal thickenings (PETs)’ when speaking about the cortical layer with cells forming thickenings in *N. caerulescens.* When speaking about cortical thickenings in other species, we will use the term ‘phi layer*’* or *‘*phi thickenings’.

Moreover, PETs in *N. caerulescens* roots are occasionally incorrectly interpreted as endodermal cell wall modifications (van de Mortel *et al.*, 2006; Hanikenne *et al.*, 2008). The confusion caused by the proximity of the two layers and deposition of additional wall material opens the question of the function of the layer with thickenings in *N. caerulescens*. In the case of phi thickenings, the barrier function is only suggested (Peterson *et al.*, 1981), but in the case of PETs it is completely unknown. To clarify whether the thickenings in *N. caerulescens* may function as an apoplasmic barrier similar to the endodermis, roots of *N. caerulescens* and *Arabidopsis thaliana* plants were tested for dye permeability. These two species have similar root anatomy (Dolan *et al.*, 1993; Zelko *et al.*, 2008), except for the thickening-forming layer in *N. caerulescens*, which is missing in *A. thaliana* roots. In the present work, using various microscopic, histochemical and spectroscopical analyses, the cell wall morphology, ultrastructure, chemical composition and development of PETs were elucidated, described and compared with the endodermal cell wall. Moreover, the differences between PETs and phi thickenings are discussed and a possible role of PETs is suggested.

## MATERIALS AND METHODS

### Plant material

Plants and seeds of *Noccaea caerulescens* were collected from a former mining site in Salzburg (Austria) and seeds were stored at 4 °C. Before germination, the seeds were washed for 5 min in commercial detergent and for 15 min in 5 % sodium hypochlorite, washed twice with distilled water and sown on 1 % pure agar media in Petri dishes. All dishes were oriented vertically in a cultivation chamber with a photoperiod of 16 h (16/8 h light:dark), 200 µmol m^−2^ s^−1^ light intensity and 25 °C. Five-day-old seedlings were fixed in 99 % methanol and stored at 4 °C. In experiments on the development of PETs, the seedlings were collected regularly every day for 5 d. Roots of plants collected directly from the locality of origin were gently washed with distilled water to remove soil particles, fixed in 99 % methanol and stored at 4 °C. For the comparative study of dye permeability to the central cylinder, plants of *Arabidopsis thaliana*, ecotype Columbia (Col-0), were cultivated in the same way as plants of *N. caerulescens* in 1 % agar media. For dye permeability experiments, living plants (without any fixation) were used.

### Cross-sectioning of roots

Cross-sections of roots were prepared using a cryomicrotome (CM3050S, Leica, Wetzlar, Germany) precooled for 24 h before sectioning to −20 °C. Root segments were collected from the middle part of the main roots, washed for 5 min in distilled water and submerged in tissue-freezing medium (Leica) in aluminium boxes (W × L × H in mm: 5 × 15 × 10). Samples were oriented in the boxes, and after freezing the boxes were unwrapped, oriented perpendicular to the direction of sectioning and stuck on the specimen chuck. Subsequently, 20-μm-thick sections were prepared.

### Visualization of PET structure by scanning and transmission electron microscopy

To visualize the details of peri-endodermal cell wall structure, segments of 5-d-old roots were separated and washed in distilled water. For scanning electron microscopy (SEM), samples were frozen in liquid nitrogen and sectioned using a cryomicrotome. Sections were fixed on aluminium stubs and dried overnight in a cryochamber at −20 °C. Subsequently, all samples were coated with a nanolayer of gold and observed using a JEOL JSM-IT300 scanning electron microscope. For transmission electron microscopy (TEM), samples were fixed for 2 h in 3 % glutaraldehyde in 0.1 m cacodylate buffer and postfixed in 0.5 % aqueous solution of osmium tetroxide overnight. After washing and dehydration in a graded ethanol and propylene oxide series, the samples were embedded in Spurr embedding medium (Serva). For a general overview, semi-thin sections were prepared and stained in 1 % (w/v) solution of Toluidine Blue O (Sigma). Approximately 70 nm ultra-thin sections were prepared using a Reichert-Jung Ultracut E ultramicrotome (Leica, Wetzlar, Germany), mounted on copper grids, and stained with 2 % uranyl acetate and 2 % lead citrate. Sections were observed using a Zeiss Libra 120 (Oberkochen, Germany) transmission electron microscope.

### Histochemical analyses of PET cell walls

For histochemical analyses, 20-μm-thick cryomicrotome sections were submerged in individual histochemical reagents. For lignin staining, 2 % phloroglucinol in 25 % HCl solution was used. For suberin staining, sections were immersed in 0.1 % (w/v) solution of Sudan Red 7B in 50 % (w/v) PEG 400 in glycerol (Brundrett *et al.*, 1991). For detection of pectins, sections were immersed in a drop of 0.05 % (w/v) Ruthenium Red. For cellulose detection, sections were immersed in a drop of 0.1 % (v/v) Calcofluor White M2R (Sigma) and subsequently one drop of 10 % KOH was added. All reagents were applied for 1–5 min; the staining solutions were then removed and the sections were rinsed thoroughly in distilled water and observed under bright-field microscopy (staining for lignin, suberin, pectins) or confocal laser scanning microscopy (autofluorescence, staining for cellulose). The autofluorescence of the tissues was observed in sections without any staining.

### Whole-mount visualization of root tissues

To visualize the whole-root distribution of endodermal cells with developed suberin lamellae and of peri-endodermal cells with thickenings, we modified the staining protocol (Lux *et al*., 2005, 2015). Fixed root samples were washed with distilled water and whole-mount-stained with 0.01 % (w/v) Fluorol Yellow 088 (Sigma–Aldrich) in lactic acid. Samples were stained for 4 h on a heating plate at 70 °C. Subsequently, after the staining solution had been washed out with distilled water, the samples were mounted in 0.1 % (w/v) FeCl_3_ solution in 50 % (v/v) glycerol and observed using a confocal laser scanning microscope.

### Immunolabelling of pectins in PETs with LM19/LM20 antibodies

For pectin immunolabelling, root segments were fixed in 4 % (w/v) paraformaldehyde in 50 mm PIPES (pH 6.9) overnight, according to Leroux *et al.* (2015). After washing in phosphate-buffered saline (PBS, pH 6.9), samples were frozen in tissue-freezing medium (Leica), sectioned with a cryomicrotome, collected in 2-mL tubes and melted in PBS solution. Thereafter, they were washed three times with PBS and incubated for 2 h in a monoclonal primary antibody to unesterified (LM19) and methyl-esterified (LM20) homogalacturonans (www.plantprobes.net) (Verhertbruggen *et al.*, 2009), with 20-fold dilution in PBS. For a negative control, primary antibody incubation was omitted. Afterwards, all sections were washed three times with PBS and incubated for 2 h in a secondary antibody (goat anti-rat IgM conjugated with fluorescein isothiocyanate; FITC, Abcam) with 50-fold dilution in PBS. After final washing in PBS, sections were post-stained with 0.1 % Calcofluor White, immersed in an antifade mounting medium (0.5 % *p*-phenylenediamine in 70 % glycerol in PBS) and observed using a confocal scanning laser microscope.

### Plasmolysis of cells with PETs

To determine whether the cytoplasm of cells with PETs is attached to these thickenings or whether there is no direct connection, roots were exposed to hypertonic water solutions of 1 m KCl and 1 m CaCl_2_ mixed at a 9:1 ratio. After exposure for 5 min, roots were observed using a bright-field microscope and plasmolysis was evaluated.

### Microscopical observations

All samples observed under bright-field microscopy were examined with a Zeiss Axioscope 2 Plus (Oberkochen, Germany). Pictures were taken with an Olympus DP-72 digital camera (Tokyo, Japan). For confocal microscopy, samples were examined with an Olympus FluoView FV1200 (Tokyo, Japan), and a ×40 or ×60 glycerol immersion lens was used. For samples stained with Calcofluor White, 405/425 nm (excitation/emission) was used, for FITC-labelled LM19 and LM20 antibodies, 473/519 nm (excitation/emission) was used. To visualize both propidium iodide and rhodamine B staining, 559/600–700 nm (excitation/emission) was used. Evaluation, image analyses and measurements were performed using ImageJ software v.1.52i (https://imagej.nih.gov/ij).

### Raman spectroscopy

To investigate the chemical composition of cell walls by Raman spectroscopy, 20-μm-thick cryotome sections were prepared, mounted in distilled water on a glass microscope slide, covered with a coverslip and sealed with nail polish to prevent water evaporation. Raman spectra were collected with a DXR Raman Microscope (Thermo Scientific, MA, USA), using a 532-nm laser and 900 lines mm^−1^ grating setup. Spectra were recorded at 10 mW laser power, using 12 s photobleaching time, 20 s acquisition time per collection and 25 collections per measurement. Spectra were collected within a spectral range of 200–3200 cm^−1^ with automatic fluorescence background correction using Omnic Atlμs software (Thermo Scientific, MA, USA). Afterwards, spectral regions outside 300–3100 cm^−1^ were cut off and the spectra were baseline-corrected (15 % coarseness), smoothed (Sawitsky-Golay, 9 points, polynomial order 4) and normalized against the highest peak within the region 2800–3100 cm^−1^ using Spectragryph version 1.0.7 (F. Menges, http://www.effemm2.de/spectragryph). Spectra were plotted using Python (version 3.5, Python Software Foundation, https://www.python.org) and are presented as means (*n* > 5).

### Comparison of root dye permeability in *N. caerulescens* and *A. thaliana*

A possible barrier function of PET was tested individually with two dyes having different modes of root tissue penetration. Propidium iodide, a highly charged molecule, is widely used as an apoplasmic tracer blocked by apoplasmic barriers like Casparian bands. Rhodamine B, a moderately lipophilic dye, is able to cross the cell membrane (Liu and Gaskin, 2004) and hence to bypass such apoplasmic barriers. Intact plants of *N. caerulescens* and *A. thaliana* were placed in separate confocal dishes with a drop of 15 μm propidium iodide or 5 μm rhodamine B dissolved in distilled water. Longitudinal optical sections were imaged immediately during the following 20 min, using a confocal laser scanning microscope. Images were taken from the root zone, where xylem vessels start to form visible thickenings, Casparian bands and PETs start to develop. The intensity of fluorescence was measured using ImageJ software, version 1.50i (https://imagej.nih.gov/ij/), as an integrated density in root tissues outside (RTO) from PETs (in *N. caerulescens*) or endodermis (in *A. thaliana*), and in root tissues inside (RTI) from endodermis (in both plants). The precise location of these tissues was specifically determined in bright-field microscopy images. The movement rate of the dye into the central cylinder and the ability of individual thickenings to influence this movement were expressed as the ratio of intensities RTI/RTO over time.

### Effect of PETs on heavy metal distribution in roots

To verify whether PETs may affect the distribution of elements in root tissues, seedlings were transferred from agar media to half-strength Hoagland solution (control) or to the same solution enriched with 500 μm ZnSO_4_ or 50 μm CdNO_3_ and kept in a cultivation chamber for 5 d. For heavy metal measurements, samples were prepared similarly to those prepared for SEM, except that a nanolayer of gold was used. Measurements were conducted using a JEOL JSM-IT300 (Tokyo, Japan) scanning electron microscope coupled with an energy-dispersive X-ray (EDX) analyser (EDAX, Ametek, PA, USA) at ×1000 magnification, 20 kV, data collection time 50 s, 10 000 counts per second, and working distance set to 11.0 mm. Data were collected from (1) outer root tissues including rhizodermis and cortex except the layer of peri-endodermis, (B) cell walls of peri-endodermis with PET, and (C) the central cylinder, mainly xylem vessels. For EDX measurements, three biological and four technical replicates were performed. All data were processed with the software TEAM Enhanced, version 4.3 (Ametek, PA,USA).

### Statistical analysis

Statistical analyses were performed using Statgraphics Centurion software (version 15.2.05).

A one-way (ANOVA) test was performed on the datasets and the statistical significance of the means difference was considered at the 0.05 probability level. For each experiment, at least four biological replicates were evaluated, if not stated otherwise.

## RESULTS

### PET structure visualization

**Fig. 1.**
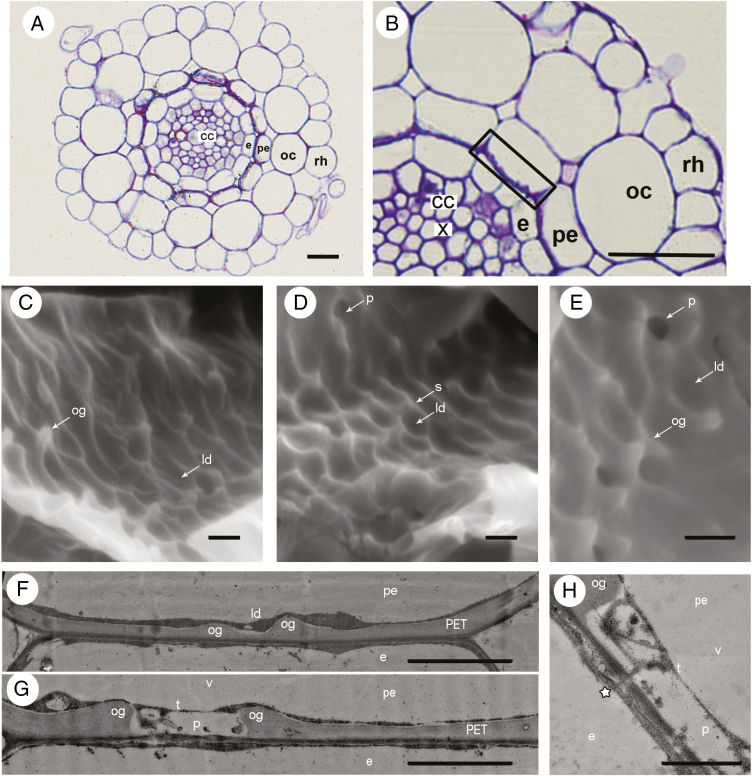
The anatomy and ultrastructure of *Noccaea caerulescens* roots show PETs. (A, B) Overview of *N. caerulescens* root stained with Toluidine Blue O showing individual cell layers: rhizodermis (rh), outer cortex (oc), peri-endodermal layer (pe) with thickenings resembling half-moon or the letter C on inner tangential cell walls, endodermis (e) and central cylinder (cc) with xylem vessels (x). The black rectangle marks the part of the cell wall depicted in the next panels. (C–H) Inner tangential cell wall of peri-endodermal cells with PETs visualized by SEM (C–E) and TEM (F–H). The relief of thickenings adjacent to the vacuole (v) and tonoplast (t) is highly structured with many outgrowths (og), local depressions (ld), shelves (s) and pores (p). The part of the peri-endodermal cell wall adjacent to the endodermal cell (e) is smooth. In the areas of peri-endodermal cells with developed PETs, plasmodesmata occur in pores (star). Scale bars: (A, B) = 20 μm; (C–H) = 2 μm.

In cross-sections of *N. caerulescens* primary roots, from the periphery to the centre the following layers can be found: root epidermis, three cortical layers including outer cortex, peri-endodermis and endodermis, pericycle and the cells of central cylinder with vascular tissues (Fig. 1A). Cells in all these layers are thin-walled, with the exception of xylem and peri-endodermal cells. The latter form typical cell wall thickenings, in cross-section resembling a half-moon or the letter C, localized on the radial and inner tangential cell walls and directly attached to the cell walls of the endodermis (Fig. 1B). According to SEM and TEM analyses (Fig. 1C–H), the cell wall material protruding towards the protoplast is massive and unevenly distributed, with a structured surface creating outgrowths, shelves, local depressions and pores. The opposite part of the cell wall facing towards the endodermal cell is smoother (Fig. 1C–E). Plasmodesmata connecting peri-endodermal and endodermal cells are present only in pores, but not in outgrowths or in local depressions (Fig. 1F–H).

### Histochemical analysis of PETs

**Fig. 2.**
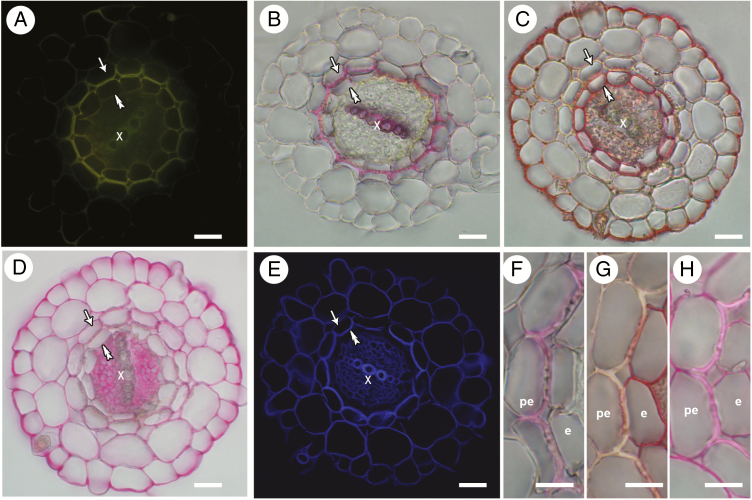
Histochemical staining of *Noccaea caerulescens* roots with marked peri-endodermal (single arrows), endodermal (double arrowheads) and xylem (x) tissues. (A) Autofluorescence of root tissues. (B) Phloroglucinol staining with visible coloration of lignified PETs and xylem vessel walls. (C) Sudan Red 7B staining showing the presence of lipidic suberin substances in endodermis and rhizodermis. (D) Ruthenium Red staining visualizing the presence of pectins. (E) Calcofluor White staining for cellulose. (F–H) Details of individual images (B–D), respectively, showing endodermal (e) and peri-endodermal (pe) cell layers. Scale bars: (A–E) = 20 μm; (F–H) = 10 μm.

The thickenings of peri-endodermal cell walls show bright autofluorescence similar to the cell walls of xylem vessels and endodermal cells (Fig. 2A), indicating the presence of phenolic substances. Histochemical analysis of root sections revealed the presence of various cell wall components (Fig. 2B–H). The pinkish reaction in PET and xylem vessel walls after phloroglucinol/HCl staining revealed the presence of lignin (Fig. 2B, F). The absence of reaction of PETs with Sudan Red 7B dye indicated the absence of suberin in the cell walls of peri-endodermal cells, in contrast to the reddish-stained cell walls of endodermal and rhizodermal cell walls (Fig. 2C, G). As opposed to lignin, a pinkish colour appearing after Ruthenium Red staining for pectin was present in all cell walls, except for the thick walls of xylem vessels and some regions of the peri-endodermal walls (Fig. 2D, H). The cellulose-binding fluorophore Calcofluor White stained all cell walls in the root sections positively (Fig. 2E).

### Raman spectroscopy

**Fig. 3:**
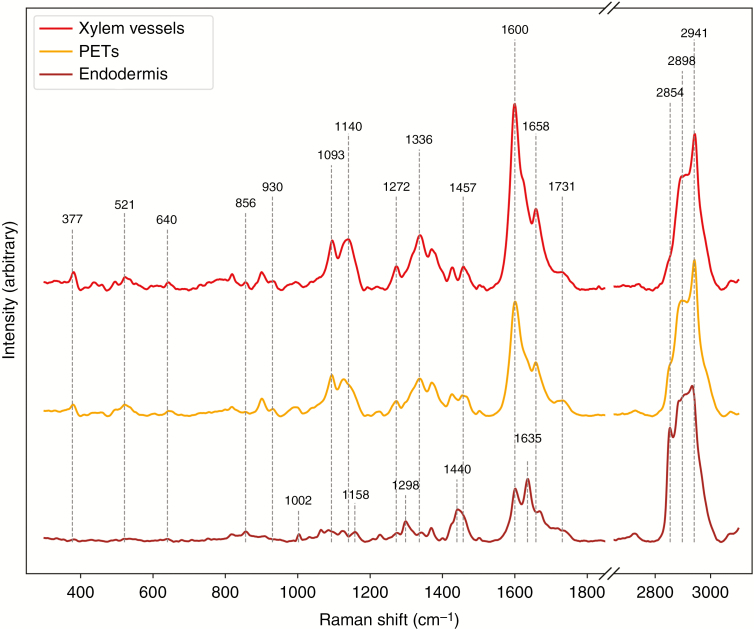
Raman spectra obtained for cell walls of individual tissues of *Noccaea caerulescens* primary roots, showing the similarities and differences in compositions of xylem vessels, PETs and endodermal cell walls.

To extend our understanding of the chemical composition of PETs, Raman spectra were obtained for the cell walls of PETs, endodermis and xylem vessels (Fig. 3). The spectrum of PETs indicated notable similarity to the xylem cell wall spectrum. Although slightly reduced in the PETs, both cell walls exhibited a relatively strong signal from phenolic components (1600 cm^−1^), especially phenolic aldehydes (1140, 1620 cm^−1^). In contrast, the endodermal spectra indicated intense suberization of the cell wall (1064, 1298, 1440, 1635 and 1700–1760 cm^−1^) and an elevated signal indicative of pectins (856 and 1700–1760 cm^−1^).

### Immunohistochemical labelling of PETs

**Fig. 4.**
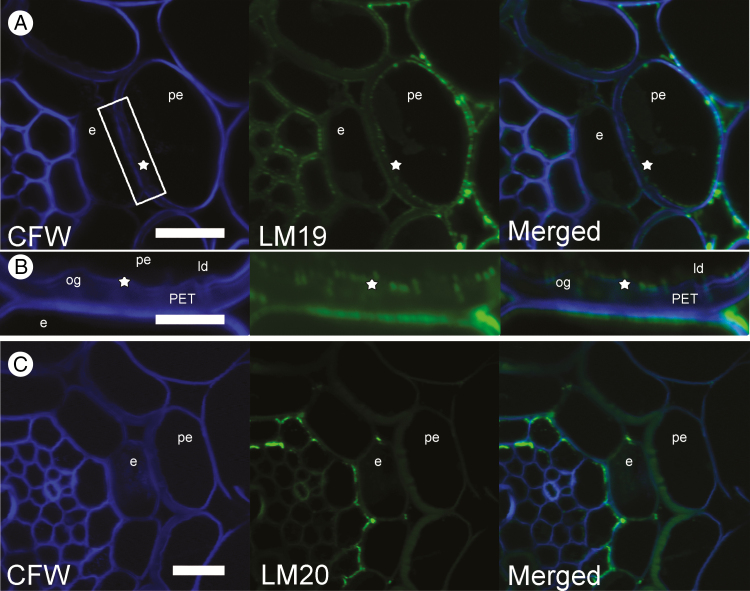
Immunolabelling of homogalacturonans distributed in cell walls of endodermal (e) and peri-endodermal (pe) tissues in *Noccaea caerulescens* roots counterstained with Calcofluor White (CFW). (A) Staining for unesterified epitopes with LM19 antibodies shows strong signal in outer peri-endodermal cell walls and inside the extracellular spaces; however, there were also epitopes located on the inner surface of peri-endodermal cells (stars). The white rectangle depicts the area magnified in (B). (B) Detail of PETs with epitopes for LM19 antibodies (stars) localized on PET outgrowths (og) or local depressions (ld). (C) Staining for methyl-esterified epitopes with LM20 shows absence of these epitopes on PET surface compared with relatively strong signal from inner endodermal cell walls. Scale bar = 15 μm.

The monoclonal antibodies LM19 and LM20, which bind to unesterified and methyl-esterified homogalacturonan domains, respectively, showed different patterns of labelling in the cell walls of the peri-endodermal layer. Epitopes for LM19 antibodies were clearly visible around the entire inner surface of peri-endodermal cells, including the inner surface of PETs and also in intercellular spaces (Fig. 4A, B). In contrast, epitopes for LM20 antibodies were absent in all cortical and peri-endodermal cells, including the surface of PETs. They were detected mainly in the cell walls of cells in the central cylinder (Fig. 4C).

### Test of PET permeability

**Fig. 5.**
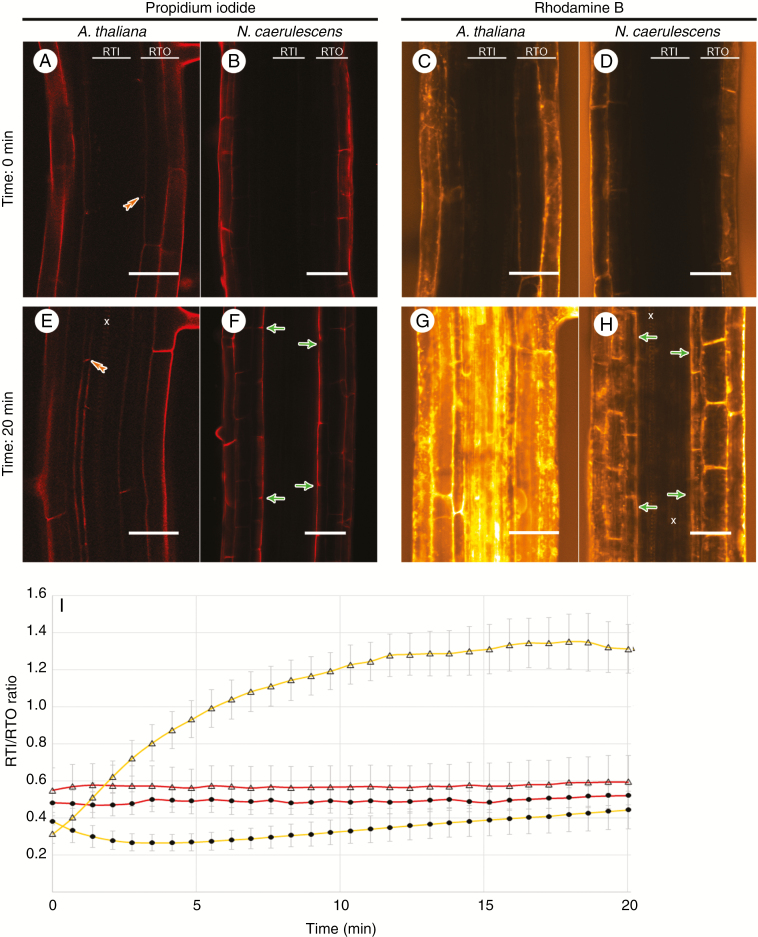
Ability of the dyes propidium iodide (A, B, E, F) and rhodamine B (C, D, G, H) to penetrate into roots of *Arabidopsis thaliana* (A, C, E, G) or *Noccaea caerulescens* (B, D, F, H) at the beginning (time 0 min) and end (time 20 min) of the experiment. Propidium iodide-stained root tissues outside (RTO) of PETs (green single arrows) or endodermis (orange double arrowheads). Root tissues inside (RTI), like xylem vessels (x), were stained only weakly (*A. thaliana*) or not at all (*N. caerulescens*). Rhodamine B stained inner tissues very intensely in *A. thaliana*, but only faintly in *N. caerulescens.* (I) Graph showing fluorescence intensities (propidium iodide, red curves; rhodamine B, yellow curves), measured in RTI and RTO areas in *A. thaliana* (triangles) and *N. caerulescens* (circles), expressed as the RTI/RTO ratio over time. Differences were evaluated by the LSD *post hoc* test (*t* = 0.05) and data are presented as mean ± s.d., with *n* = 8. Scale bar = 50 μm.

The ability of fluorescent dyes to penetrate roots with or without PETs was tested in *N. caerulescens* and also in *A. thaliana* roots (Fig. 5A–I). In both species, propidium iodide entered the apoplasm of epidermal and cortical cells almost immediately; its movement was blocked by Casparian bands in *A. thaliana* (Fig. 5A, E) and by PETs in *N. caerulescens* roots (Fig. 5B, F). Only a very low amount of the dye penetrated to the central cylinder and stained xylem vessels. Rhodamine B, being able to penetrate both apoplasm and symplasm, entered the *A. thaliana* root very quickly, and markedly stained tissues across the whole root (Fig. 5C, G). In the case of *N. caerulescens*, the dye entered the cell walls and protoplasts in cells peripherally from PETs, leaving the central cylinder tissues stained only slightly (Fig. 5D, H). These observations were confirmed by the ratio of fluorescence intensities measured in RTO from PETs (*N. caerulescens*) or endodermis (*A. thaliana*), and in RTI from the endodermis (in both plants) (Fig. 5I).

### Origin, development and distribution of PETs in the root system of *N. caerulescens* plants

**Fig. 6.**
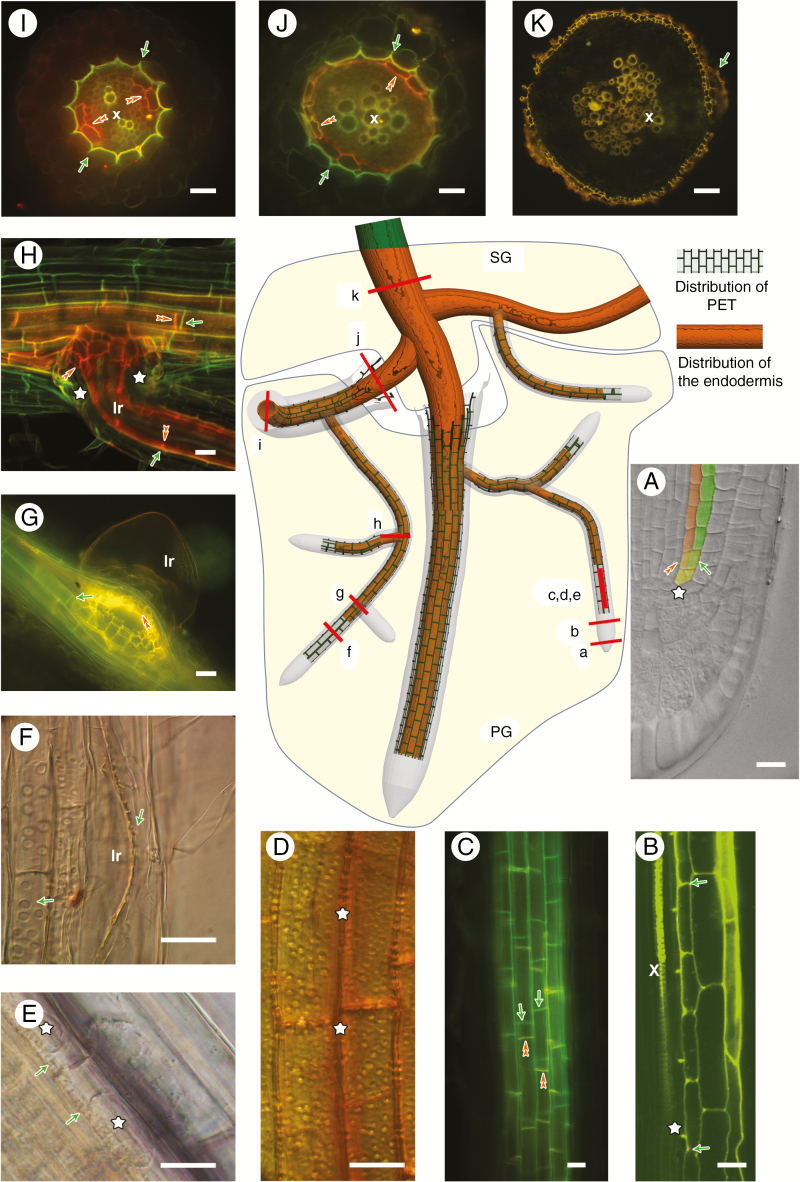
Scheme of distribution of PETs and suberized cells of endodermis in the root system of *Noccaea caerulescens*. The scheme of the root system shown in the centre of the figure visualizes a young, primary growing (PG zone) root with developed PETs (grey rectangles) and suberized endodermis (orange colour). After the onset of secondary growth (SG zone), PETs together with rhizodermis and the rest of the primary cortex are displaced from the root and the root remains covered by suberized cells of the periderm. Red lines marked with letters (a–k) represent the cross-sections through individual regions of the root system shown in the images (A–K) around the central scheme. In (A–K) green arrows indicate PETs and orange double arrowheads indicate endodermis. (A) Optical section through the apical meristem showing the common origin of peri-endodermal and endodermal cells. After initial division (star), two layers appear, the future peri-endodermis located centrifugally (green layer) and the future endodermis located centripetally (orange layer). (B) Optical section showing the formation of PETs in the proximity of forming xylem vessels (x) and Casparian bands (star). (C) Fully developed cells with PETs visible in a longitudinal view as regular rectangles with a ladder-like shape. (D) Inner tangential surface of developed cells with PETs is structured with many pores. Pores on the radial cell walls (star) are localized in the same position as pores in the adjacent cell. (E) Plasmolysis of peri-endodermal cells shows detachment of the protoplasts (star) from thickened cell walls (arrows). (F) During lateral root (lr) development, a cylindrical network of cells with PETs disintegrates and cells are forced and displaced outwards. (G) A gap in the cylindrical network of cells with PETs caused by the growing lateral root (lr) is never sealed with newly formed thickenings. In contrast, the cells of the endodermis suberize in this place and create a protective collar around the opening. (H) Interconnection of the primary and lateral root (lr) shows missing PETs at the site of connection (stars) and the presence of a protective collar of suberized endodermal cells. (I) Cross-section through a young root before secondary growth with fully developed PETs in all cells of the peri-endodermis. Centripetally towards the central cylinder with xylem vessels (x), some of the endodermal cells are suberized. (J) Cross-section through a root after onset of secondary growth. Growing tissues emboss outer cells with PETs. (K) Cross-section of an old, secondarily thickened root surrounded by layers of suberized cells. Only remnants of cells with PETs are attached to them. Scale bar = 20 μm.

After germination, PETs start to form in roots ~4.5–5 mm long (4-d-old seedlings) at 1–1.5 mm from the root–shoot junction. Formation proceeds towards the root tip, but never towards the junction. At the same time, the endodermis starts to suberize progressively from the root–shoot junction towards the root tip. Both the peri-endodermal cell layer forming the PET and the neighbouring cell layer forming the endodermis share the same initial cell. During meristematic division and tissue organization in the root meristematic zone, the innermost cortical cell layer divides periclinally. After this division, the centrifugally located layer produces a row of cells forming the PET, while the centripetally located cell layer develops into the endodermis (Fig. 6A). In the case of PETs, cell wall material is firstly deposited into the corners between two peri-endodermal cells, and only later onto the inner tangential cell wall (Fig. 6B). Fully developed cells with PETs resemble prolonged rectangles in longitudinal view (Fig. 6C). The radial and inner tangential cell walls develop the typical relief made up of outgrowths and pores of various shapes and sizes. The position of pores on the inner tangential wall is random, but pores on the radial cell walls are adjacent to pores in the neighbouring cells and are possibly interconnected (Fig. 6D). The cytoplasm is not attached to these thickenings (as it is in case of Casparian bands), and it is visibly detached during plasmolysis (Fig. 6E). The developed cells with PETs form a network of cylindrical shape around the central cylinder, with individual cells being tightly attached to each other without intercellular spaces. An exception to this layout is the formation of lateral roots. During this process, cells with PETs are displaced by growing root primordia, the cylindrical network of PETs is open and the continuity of the network is disrupted (Fig. 6F, G). This contrasts with the behaviour of the suberized endodermal layer, which is, after the outgrowth of a lateral root, sealed with a group of newly formed small suberized cells, creating a protective collar around the lateral root base. This collar interconnects the suberized endodermal network between the main and lateral roots. The cells of the peri-endodermis never form PETs in the collar zone of a new lateral root (Fig. 6G, H).

Except for the lateral root formation described above, PETs surround the inner root tissues as a complete cylinder during the entirety of primary root growth (Fig. 6I). After the onset of secondary growth, the internal secondary meristems extend the root diameter and the peripheral cells of rhizodermis and cortex (including the peri-endodermal layer) disrupt and disintegrate (Fig. 6J). The newly formed cells of the periderm suberize similarly to endodermal cells, and the cells of the peri-endodermis with PETs remain only occasionally as remnants on the root surface (Fig. 6K).

### Effect of PETs on heavy metal distribution

**Fig. 7.**
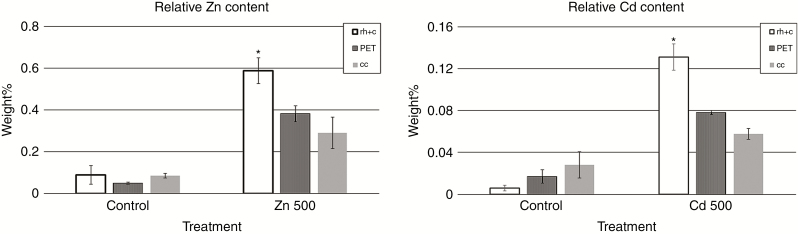
Relative contents of Zn and Cd expressed as weight percentage in individual tissues of *Noccaea caerulescens* roots after exposure of plants to control (0 Zn, 0 Cd), 500 μm ZnSO_4_ (Zn 500) or 50 μm CdNO_3_ (Cd 50). Data were collected from outer root tissues composed of rhizodermis and cortex (rh+c), cell walls of PETs and the central cylinder (cc). *P < 0.05 between tissues in one treatment.

Peri-endodermal thickenings as massive structures may affect the movement of fluorescent dyes as well as the distribution of elements across the root. EDX analyses revealed the highest amount of Zn and Cd in cells of the outer root tissues (rhizodermis and cortical layer). This is in contrast with significantly lower amounts of these elements localized in PETs, and even less Zn and Cd was localized in the central cylinder tissues (xylem vessels) (Fig. 7).

## DISCUSSION

Roots of *N. caerulescens* develop a specific cell layer with unusual cell wall thickenings, the peri-endodermis. Although the thickenings may resemble type-I phi thickenings, i.e. in cells adjacent to the endodermis (van Tieghem, 1888), they are different from those described in various species and do not resemble the Greek letter phi but rather form a half-moon shape or the shape of the letter C. Additionally, our data suggest that there are more differences between common phi thickenings and C-shaped thickenings in *N. caerulescens.* Thickenings in *N. caerulescens* roots are formed in the cell layer adjacent to the endodermis, and these two layers share a common origin. This is in general valid for the phi thickenings (Esau, 1943). In apple tree (*Pyrus malus*) roots, Mackenzie (1979) positioned this initial cell division 550–800 μm from the root tip. In *N. caerulescens*, the initial cell division appeared already ~100–150 µm behind the root–cap boundary. The differentiation of phi thickenings occurs in other species at different distances (Mackenzie, 1979; Pratikakis *et al.*, 1998; Soukup *et al.*, 2004; Pan *et al.*, 2006). The thickenings in *N. caerulescens* are fully developed at the distance of 400–600 μm, close to the protoxylem differentiation zone (Zelko *et al.*, 2008), where endodermal cells have already developed Casparian bands. Based on our histochemical and spectroscopic investigations, PETs in *N. caerulescens* roots are composed mainly of cellulose and of phenolic compounds typical of lignin polymers. These components are present both in the thickenings and in xylem vessels. Mature endodermal cell walls show a rather different chemical composition, dominated by suberin. Similarly, many phi thickenings in other species can be distinguished from endodermal cells by the presence of both cellulose and lignin (Haas *et al.*, 1976; Mackenzie, 1979; Zelko *et al.*, 2008; Fernandez-Garcia *et al.*, 2009, 2014; Idris and Collings, 2015) and the absence of suberin (Peterson *et al.*, 1981; Pratikakis *et al.*, 1998; Idris and Collings, 2015). Surprisingly, the immunodetection of pectic substances revealed the presence of homogalacturonan epitopes on the cell wall surface facing the lumen. The combination of lignin and pectin polymers in one cell wall is not common, as lignification is related mainly to secondary cell wall development, while pectins are reduced or completely missing in this cell wall type (Willats *et al.*, 2001).

In their further development, the thickenings in *N. caerulescens* behave comparably to phi thickenings in other plants. When new lateral roots are formed, the cortical layers are disrupted to allow their growth. During this process, cortical cells with thickenings in *N. caerulescens* are displaced until a fissure is created between them. In the case of phi thickenings in apple tree, cells with thickenings above the lateral root primordia are completely pushed out until an opening is created. Later, a new cell layer with phi thickenings is formed in the lateral root without attachment to the phi thickenings in the main root (Weerdenburg and Peterson, 1983). We observed the same process in roots of *N. caerulescens*, where direct connections between the thickenings in the main and lateral roots are missing, contrary to endodermal cells interconnected between the main and lateral roots through the suberized collar zone. Cells with PETs remain alive even after cell wall lignification. During plasmolysis, the plasma membrane is detached from the wall. This situation is comparable with the detachment of the plasma membrane from phi thickenings in *Pelargonium hortorum* (Haas *et al.*, 1976).

After the onset of secondary growth, during periderm formation, the root increases its diameter and all the peripheral tissues are shed. Cell layers with thickenings are also broken and finally only some remnants may be visible on the root surface. This situation is common also for phi thickenings in general and is described in other species as well (Mackenzie, 1979; Weerdenburg and Peterson, 1983; Nii *et al.*, 2004; Soukup *et al.*, 2004).

Thickenings similar to those described in roots of *N. caerulescens* can be found in red bayberry (*Myrica rubra*) roots; they are randomly distributed in various cortical cells and described as ‘crescent-like’ (Song *et al.*, 2011). Even if it is not mentioned explicitly, these thickenings are probably sloughed off during secondary growth, similar to the phi thickenings. A different scenario can be observed in the roots of some Myrtaceae species, where crescent thickenings are formed in the primary cortex and are shed during secondary growth, but a multilayered polyderm is formed with new layers of alternating endodermis-like cells and cells with crescent thickenings (Tuladhar *et al*., 2014, 2015; Tuladhar and Nii, 2017). Moreover, the cells with thickenings in the polyderm are produced inside the endodermis by the mitotic activity of the pericycle (Nii *et al.*, 2012), unlike the phi thickenings outside the endodermis, which are formed by the activity of root initials. Despite the similar shape of the thickenings in the above-mentioned roots, from the published images it is not possible to recognize any outgrowths or pores like those on the cell walls of *N. caerulescens* roots.

Generally, the formation of cell wall thickenings is a mechanism that has a protective role. If material is selectively deposited onto one area of the cell wall, massive cell wall outgrowths can be produced. In the case of fungal or bacterial attack, appositions or papillae composed of various substances (cellulose, lignin, callose, pectins) are formed in the place of infection (Yedidia *et al.*, 1990; Collinge, 2009; Leroux *et al.*, 2011). A localized encasement of the penetrating pathogen into cell wall material should eliminate the harmful effect on the plant cell and improve cell wall resistance. The orientation of these thickenings and outgrowths depends on the fungal attack and is not strictly localized to one cell wall.

Warts and vestures, which are kinds of wall thickenings or outgrowths (Ohtani *et al.*, 1983), are also formed on the inner side of lignified secondary cell walls in some trees. Warts are simple outgrowths that are formed in lignifying parenchyma cells, fibres and tracheids. Vestures are larger and more complex outgrowths, formed mainly around bordered pits in fibres, tracheids and xylem vessels (Liese, 1963; Meylan and Butterfield, 1974; Parameswaran and Liese, 1977). Compared with warts, the role of vestures is relatively well known. They provide mechanical support to pit membranes and regulate the degree of membrane deflection. This prevents membrane rupture, increases resistance to embolism and decreases the probability of air-seeding between vessels (Choat *et al.*, 2004; Jansen *et al.*, 2004). Warts grow gradually to their final stage, followed by cell autolysis (Baird *et al.*, 1974). This is similar to PETs, although when compared with vessels, peri-endodermal cells are living, even after the formation of thickenings ends. Ray cells associated with xylem vessels in trees are likewise living, even after lignification. They are in direct contact with vessels and have thick lignified cell walls with numerous pits or pores, but their protoplast is living. Moreover, a non-lignified protective layer containing low- and high-methyl-esterified pectins (Plavcová and Hacke, 2011) is deposited between the cell wall and plasma membrane (Murakami *et al.*, 1999). Although this protective layer has many suggested functions, the most promising idea, resulting from our comparison with *N. caerulescens* thickenings, is its role in preserving apoplasmic continuity. The movement of material into or out of the ray cell is not restricted solely to the plasma membrane facing the pit or pore in the cell wall, but, thanks to the pectinaceous protective layer, the whole protoplast surface is in contact with apoplasmic space (Barnett *et al.*, 1993). The presence of pectins in a protective layer is similar to the pectins at the inner surface of thick cell walls in *N. caerulescens* roots. Whether these thickenings have a role in material transport similar to that proposed for ray cells remains to be solved. Other types of cells with a living protoplast and forming outgrowths are transfer cells in various species, facilitating fast transfer of solutes (Talbot *et al.*, 2002). Contrary to cell walls in transfer cells, PETs are not so structured and extended. Moreover, massive deposition of lignin into the cell walls of PETs will more inhibit than facilitate solute transport.

As verified by our experiments using fluorescent dyes, PETs appear to have some negative effect on solution flow. In the case of *A. thaliana* roots without any PETs, propidium iodide was not able to enter the central cylinder because of the apoplasmic blockage caused by Casparian bands. In the same species, this barrier was not sufficient to block the lipophilic dye rhodamine B, which also moves across cell membranes. On the contrary, in *N. caerulescens* roots both dyes were blocked by PETs without any staining of endodermal cells. As these data suggest, PETs are able to block both dyes and may serve not only as an apoplasmic but partially also as a symplasmic barrier, or at least as some kind of retardant or sorting border. The slowdown and sorting could be facilitated by the restriction of the flow of solutes to plasmodesmata in cell wall pores.

Moreover, we need to take into account that *N. caerulescens* is a heavy-metal-tolerant hyperaccumulating plant; therefore, we need to consider an additional role of PETs in this species. Nedelkoska and Doran (2000) observed a 7- to 10-d-long retention of cadmium in a cell wall fraction of *N. caerulescens*. Only after that time was heavy metal released to the symplasm. Our measurements using EDX spectroscopy also revealed preferential accumulation of Zn and Cd in cortical tissues and significantly less was localized in cell walls of peri-endodermis or xylem vessels. The view of PETs as a low-permeable, highly lignified structure support these findings. However, total blockage of all metal transport through PETs should not be expected, as we measured some content of metal also in xylem vessels, and the localization of NcNramp1, a transporter of Cd in *N. caerulescens*, indicates the presence of these proteins in endodermal cell walls (Milner *et al.*, 2014). It means some route for the passage of Cd through PETs to transporters on endodermal cells must exist. Metal transport and utilization are also a potential source of reactive oxygen species. It is possible that these thickenings may trap the released radicals and use them for lignification, since radical coupling is a necessary step for lignin polymerization (Wang *et al.*, 2013).

Based on their position, chemical composition and development in roots, the PETs in *N. caerulescens* roots resemble the phi thickenings present in roots of many plant species. On the other hand, the specific C-shape, the massive deposition of cell wall material, the presence of a pectinaceous layer and the barrier ability of PETs are features not typical of phi thickenings. Interestingly, a mainly barrier function is a trait typical of endodermal cells, which do not share many similarities with the peri-endodermal cell layer or the PET itself. Therefore, our recent findings point to a specific role that the peri-endodermal layer may play in roots of hyperaccumulating *N. caerulescens* plants.

## References

[CIT0001] Aleamotu’a M, Tai Y-T, McCurdy D, Collings D 2018 Developmental biology and induction of phi thickenings by abiotic stress in roots of the Brassicaceae. Plants 7: 47. 10.3390/plants7020047 PMC6027303 29921823

[CIT0002] Baird WM, Parham RA, Johnson MA 1974 Development and composition of the warty layer in balsam fir. I. Development. Wood Fiber 6: 114–125.

[CIT0003] Barnett JR, Cooper J, Bonner LJ 1993 The protective layer as an extension of the apoplast. IAWA Journal 14: 163–171.

[CIT0004] Broadley MR, White PJ, Hammond JP, Zelko I, Lux A 2007 Zinc in plants. New Phytologist 173: 677–702. 17286818 10.1111/j.1469-8137.2007.01996.x

[CIT0005] Brundrett MC, Kendrick B, Peterson CA 1991 Efficient lipid staining in plant material with Sudan Red 7B or Fluorol Yellow 088 in polyethylene glycol-glycerol. Biotechnic & Histochemistry 66: 111–116. 1716161 10.3109/10520299109110562

[CIT0006] Choat B, Jansen S, Zwieniecki MA, Smets E, Holbrook NM 2004 Changes in pit membrane porosity due to deflection and stretching: the role of vestured pits. Journal of Experimental Botany 55: 1569–1575. 15181107 10.1093/jxb/erh173

[CIT0007] Collinge DB 2009 Cell wall appositions: the first line of defence. Journal of Experimental Botany 60: 351–352. 19204034 10.1093/jxb/erp001

[CIT0008] Dolan L, Janmaat K, Willemsen V, et al 1993 Cellular organisation of the *Arabidopsis thaliana* root. Development 119: 71–84. 8275865 10.1242/dev.119.1.71

[CIT0009] Esau K 1943 Vascular differentiation in the pear root. Hilgardia 15: 299–324.

[CIT0010] Fernandez-Garcia N, Lopez-Perez L, Hernandez M, Olmos E 2009 Role of phi cells and the endodermis under salt stress in *Brassica oleracea*. New Phytologist 181: 347–360. 19121032 10.1111/j.1469-8137.2008.02674.x

[CIT0011] Fernández-García N, López-Berenguer C, Olmos E 2014 Role of phi cells under abiotic stress in plants. In: Morte A, Varma A, eds. Root engineering. Berlin: Springer, 23–37.

[CIT0012] Gerrath JM, Covington L, Doubt J, Larson DW 2002 Occurrence of phi thickenings is correlated with gymnosperm systematics. Canadian Journal of Botany 80: 852–860.

[CIT0013] Guttenberg von H 1968 Der primäre Bau der Angiospermenwurzel. Berlin: Borntraeger.

[CIT0014] Haas DL, Carothers ZB, Robbins RR 1976 Observations on the phi-thickenings and Casparian strips in *Pelargonium* roots. American Journal of Botany 63: 863–867.

[CIT0015] Hanikenne M, Talke IN, Haydon MJ, et al 2008 Evolution of metal hyperaccumulation required *cis*-regulatory changes and triplication of HMA4. Nature 453: 391–395. 18425111 10.1038/nature06877

[CIT0016] Idris NA, Collings DA 2015 The life of phi: the development of phi thickenings in roots of the orchids of the genus *Miltoniopsis*. Planta 241: 489–506. 25377920 10.1007/s00425-014-2194-z

[CIT0017] Jansen S, Baas P, Gasson P, Lens F, Smets E 2004 Variation in xylem structure from tropics to tundra: evidence from vestured pits. Proceedings of the National Academy of Sciences of the USA 101: 8833–8837. 15163796 10.1073/pnas.0402621101 PMC423281

[CIT0018] Leroux O, Leroux F, Bagniewska-Zadworna A, et al 2011 Ultrastructure and composition of cell wall appositions in the roots of *Asplenium* (Polypodiales). Micron 42: 863–870. 21708469 10.1016/j.micron.2011.06.002

[CIT0019] Leroux O, Sørensen I, Marcus SE, Viane RL, Willats WG, Knox JP 2015 Antibody-based screening of cell wall matrix glycans in ferns reveals taxon, tissue and cell-type specific distribution patterns. BMC Plant Biology 15: 56. 25848828 10.1186/s12870-014-0362-8 PMC4351822

[CIT0020] Liese W 1963 Tertiary wall and warty layer in wood cells. Journal of Polymer Science Part C: Polymer Symposia 2: 213–229.

[CIT0021] Liu Z, Gaskin RE 2004 Visualisation of the uptake of two model xenobiotics into bean leaves by confocal laser scanning microscopy: diffusion pathways and implication in phloem translocation. Pest Management Science 60: 434–439. 15154509 10.1002/ps.816

[CIT0022] Lopez‐Perez L, Fernandez‐Garcia N, Olmos E, Carvajal M 2007 The phi thickening in roots of broccoli plants: an acclimation mechanism to salinity? International Journal of Plant Sciences 168: 1141–1149.

[CIT0023] Lux A, Luxová M, Abe J, Morita S 2004 Root cortex: structural and functional variability and responses to environmental stress. Root Research 13: 117–131.

[CIT0024] Lux A, Morita S, Abe J, Ito K 2005 An improved method for clearing and staining free-hand sections and whole-mount samples. Annals of Botany 96: 989–996. 16192293 10.1093/aob/mci266 PMC4247103

[CIT0025] Lux A, Vaculík M, Kováč J 2015 Improved methods for clearing and staining of plant samples. In: Yeung ECT, Stasolla C, Sumner MJ, Huang BQ, eds. Plant microtechniques and protocols. Cham: Springer International, 167–178.

[CIT0026] Mackenzie KAD 1979 The development of the endodermis and phi layer of apple roots. Protoplasma 100: 21–32.

[CIT0027] Meylan BA, Butterfield BG 1974 Occurrence of vestured pits in the vessels and fibres of New Zealand woods. New Zealand Journal of Botany 12: 3–18.

[CIT0028] Milner MJ, Mitani-Ueno N, Yamaji N, et al 2014 Root and shoot transcriptome analysis of two ecotypes of *Noccaea caerulescens* uncovers the role of *NcNramp1* in Cd hyperaccumulation. Plant Journal 78: 398–410. 24547775 10.1111/tpj.12480

[CIT0029] van de Mortel JE, Villanueva LA, Schat H, et al 2006 Large expression differences in genes for iron and zinc homeostasis, stress response, and lignin biosynthesis distinguish roots of *Arabidopsis thaliana* and the related metal hyperaccumulator *Thlaspi caerulescens*. Plant Physiology 142: 1127–1147. 16998091 10.1104/pp.106.082073 PMC1630723

[CIT0030] Murakami Y, Funada R, Sano Y, Ohtani J 1999 The differentiation of contact cells and isolation cells in the xylem ray parenchyma of *Populus maximowiczii*. Annals of Botany 84: 429–435.

[CIT0031] Nedelkoska TV, Doran PM 2000 Hyperaccumulation of cadmium by hairy roots of *Thlaspi caerulescens*. Biotechnology and Bioengineering 67: 607–615. 10649235 10.1002/(sici)1097-0290(20000305)67:5<607::aid-bit11>3.0.co;2-3

[CIT0032] Nii N, Ohtsuka S, Ye L, Song Y 2012 Formation of endodermis-like cells with Casparian strip and thick wall cells derived from pericycle in the roots of *Feijoa sellowiana* (Myrtaceae). Journal of the Japanese Society for Horticultural Science 81: 314–319.

[CIT0033] Nii N, Pan CX, Ogawa Y, Cui S 2004 Anatomical features of the cell wall ingrowth in the cortical cells outside the endodermis and the development of the Casparian strip in loquat trees. Engei Gakkai zasshi 73: 411–414.

[CIT0034] Ohtani J, Meylan BA, Butterfield BG 1983 Occurrence of warts in the vessel elements and fibres of New Zealand woods. New Zealand Journal of Botany 21: 359–372.

[CIT0035] Pan CX, Nakao Y, Nii N 2006 Anatomical development of phi thickening and the Casparian strip in loquat roots. Journal of the Japanese Society for Horticultural Science 75: 445–449.

[CIT0036] Parameswaran N, Liese W 1977 Occurrence of warts in bamboo species. Wood Science and Technology 11: 313–318.

[CIT0037] Peterson CA, Emanuel ME, Weerdenburg CA 1981 The permeability of phi thickenings in apple (*Pyrus malus*) and geranium (*Pelargonium hortorum*) roots to an apoplastic fluorescent dye tracer. Canadian Journal of Botany 59: 1107–1110.

[CIT0038] Plavcová L, Hacke UG 2011 Heterogeneous distribution of pectin epitopes and calcium in different pit types of four angiosperm species. New Phytologist 192: 885–897. 21801182 10.1111/j.1469-8137.2011.03842.x

[CIT0039] Pratikakis E, Rhizopoulou S, Psaras GK 1998 A phi layer in roots of *Ceratonia siliqua* L. Botanica Acta 111: 93–98.

[CIT0040] Richau KH, Kozhevnikova AD, Seregin IV, et al 2009 Chelation by histidine inhibits the vacuolar sequestration of nickel in roots of the hyperaccumulator *Thlaspi caerulescens*. New Phytologist 183: 106–116. 19368671 10.1111/j.1469-8137.2009.02826.x

[CIT0041] Russow E 1875 Betrachtungen über das Leitbündel- und Grundgewebe aus vergleichend morphologischem und phylogenetischem Gesichtspunkt. Dorpat: Druck von Schnakenburg’s litho- und typogr. Anstalt.

[CIT0042] Song Y, Ye L, Nii N 2011 Effects of soil water availability on development of suberin lamellae in the endodermis and exodermis and on cortical cell wall thickening in red bayberry (*Myrica rubra* Sieb. et Zucc.) tree roots. Scientia Horticulturae 129: 554–560.

[CIT0043] Soukup A, Malá J, Hrubcová M, et al 2004 Differences in anatomical structure and lignin content of roots of pedunculate oak and wild cherry-tree plantlets during acclimation. Biologia Plantarum 48: 481–489.

[CIT0044] Talbot MJ, Offler CE, McCurdy DW 2002 Transfer cell wall architecture: a contribution towards understanding localized wall deposition. Protoplasma 219: 197–209. 12099220 10.1007/s007090200021

[CIT0045] van Tieghem P 1888 Le réseau de soutien de l’écorce de la racine. Annales des Sciences Naturelles, Botanique 7: 375–378.

[CIT0046] Tuladhar A, Nii N 2017 Anatomical studies on *Myrtaceae* roots. Acta Horticulturae 1166: 55–62.

[CIT0047] Tuladhar A, Ohtsuka S, Nii N 2014 Formation of exclusive pattern during accumulation of ligno-suberic material in cell wall of Myrtaceae root tissues including epidermis, exodermis, endodermis and polyderm. Plant Root 8: 24–32.

[CIT0048] Tuladhar A, Ohtsuka S, Nii N 2015 Anatomical study on wax apple (*Syzygium samarangense*) roots under long-term water-logged conditions. Tropical Agriculture and Development 59: 1–6.

[CIT0049] Verhertbruggen Y, Marcus SE, Haeger A, Ordaz-Ortiz JJ, Knox JP 2009 An extended set of monoclonal antibodies to pectic homogalacturonan. Carbohydrate Research 344: 1858–1862. 19144326 10.1016/j.carres.2008.11.010

[CIT0050] Wang Y, Chantreau M, Sibout R, Hawkins S 2013 Plant cell wall lignification and monolignol metabolism. Frontiers in Plant Science 4: 1–14. 23847630 10.3389/fpls.2013.00220 PMC3705174

[CIT0051] Weerdenburg CA, Peterson CA 1983 Structural changes in phi thickenings during primary and secondary growth in roots. 1. Apple (*Pyrus malus*) Rosaceae. Canadian Journal of Botany 61: 2570–2576.

[CIT0052] Willats WG, McCartney L, Mackie W, Knox JP 2001 Pectin: cell biology and prospects for functional analysis. Plant Molecular Biology 47: 9–27. 11554482

[CIT0053] Yedidia I, Benhamou N, Chet I 1990 Induction of defense responses in cucumber plants (*Cucumis sativus* L.) by the biocontrol agent *Trichoderma harzianum*. Applied and Environmental Microbiology 65: 1061–1070. 10.1128/aem.65.3.1061-1070.1999 PMC91145 10049864

[CIT0054] Zelko I, Lux A, Czibula K 2008 Difference in the root structure of hyperaccumulator *Thlaspi caerulescens* and non-hyperaccumulator *Thlaspi arvense*. International Journal of Environment and Pollution 33: 123–132.

